# Editorial: Balancing alloantigen-induced immune responses and anti-tumor immunity in transplantation

**DOI:** 10.3389/fimmu.2025.1663706

**Published:** 2025-07-28

**Authors:** Rita Maccario, Marcello Maestri, Luigi Nespoli

**Affiliations:** ^1^ Cell Factory, Pediatric Hematology/Oncology Lab, Fondazione IRCCS Policlinico San Matteo, Pavia, Italy; ^2^ Department of Clinical-Surgical, Diagnostic and Pediatric Sciences, Universita di Pavia, Pavia, Italy; ^3^ Department of Pediatric Science, Universita degli Studi dell’Insubria, Varese, Italy

**Keywords:** alloimmunity, transplantation, immune tolerance, immunosuppression, cell-based therapies, opportunistic infections

## Introduction

Transplantation medicine has transformed from an experimental procedure with limited success to a life-saving intervention performed hundreds of thousands of times annually worldwide. Despite remarkable advances in unraveling the immune cascades and molecular interactions involved in solid organ and hematopoietic stem cells transplantation (HCT), alloimmune reactions remain the primary barrier to optimal long-term transplant outcomes.

Extensive research has established the pivotal role of T-cell-mediated and antibody-mediated adaptive immunity in alloimmune reactions. More recently, the critical role of innate immunity in early alloreactive response induction and maintenance of chronic organ rejection and chronic graft-versus-host disease (GVHD) has gained recognition (1, 2). The complexity of alloimmune reactions continues to challenge the transplant community. While acute solid organ rejection rates have declined significantly over the past three decades, improvements in long-term graft survival have plateaued. This disconnect underscores our incomplete understanding of the chronic alloimmune processes that ultimately lead to graft failure.

The allorecognition landscape metaphor proposed by Rinkevich bridges natural and clinical transplantation by conceptualizing self/non-self-recognition as dynamic continuum of evolving states that shift throughout a patient’s lifespan. Rinkevich’s innovative perspective suggests that immunity evolved not as a pathogen-driven mechanism but as a system to preserve individuality by preventing invasion from conspecific cells. This revolutionary concept provides valuable insight, advocating for reevaluation of fundamental immune tolerance principles derived from natural allorecognition mechanisms observed in marine invertebrates and humans, such as tolerogenic fetal-maternal immune interactions during successful fetal implantation. These biological insights could inspire novel therapeutic strategies for achieving sustained graft acceptance.

## Strategies to induce donor-recipient tolerance

Achieving long-lasting donor-recipient tolerance requires complex interventions before, during, and after transplantation. Primary considerations include identifying optimal donor-recipient matches regarding major and minor histocompatibility antigens, developing predictive biomarkers for post-transplant graft rejection or GVHD onset, and determining optimal immunosuppressive therapy to prevent or mitigate graft rejection or GVHD while preserving adequate anti-pathogen and anti-tumor immune surveillance.

Haider et al. addressed a critical need in transplantation immunology by achieving high-resolution HLA genotyping with enhanced accuracy and efficiency. This advancement is highly relevant for improving donor-recipient matching and reducing alloimmune complications.

Pasi et al. explored the underexamined role of recipient-specific anti-HLA antibodies (RSA) in HCT, investigating RSA-mediated damage mechanisms and their potential involvement in endothelial damage contributing to GVHD. Particular attention was directed toward RSA targeting non-inherited maternal or paternal antigens in haploidentical HCT (3).

Tao et al. provided detailed exploration of TIM proteins and miRNA in transplantation immunity, specifically focusing on liver, kidney, and heart transplantation. Their work examined immune response regulation by TIM proteins and highlighted miRNA influence on transplantation outcomes, including the miRNA-TIM network with potential for improving transplantation results.

Luo et al. identified two distinct MICA polymorphism types that differentially regulate NKG2D receptor activation on NK lymphocytes. Given NK alloreactivity’s role in transplantation, these findings suggest that genetic variation in MICA may contribute to understanding individual differences in post-transplant alloreactive immune activation.

Naciri Bennani et al. explored innovative approaches to overcoming alloimmune barriers such as ABO incompatibility, reporting favorable outcomes in ABO-incompatible kidney transplant recipients, including those with exceptionally high baseline antibody titers. These successful outcomes required aggressive immunosuppression, indicating both feasibility and inherent challenges in extending transplant eligibility.

## Opportunistic infections in transplantation

Achieving favorable long-term outcomes in HCT or solid organ transplantation depends critically on balancing effective prevention of severe GVHD and allograft rejection with risks of compromised anti-pathogen and anti-tumor immune surveillance. Powerful immunosuppressive drugs that substantially reduce rejection and GVHD incidence increase transplant recipient susceptibility to life-threatening opportunistic infections.

Addressing heightened infection risk from intensive immunosuppression, Zhong et al. evaluated prophylactic CMV hyperimmune globulin (CMV-Ig) effectiveness, demonstrating significant reductions in human CMV (HCMV) viremia and improved renal function preservation in ABO-incompatible transplant recipients. Their findings advocate incorporating passive immune prophylaxis in high-risk management protocols.

Cuesta-Martín de la Cámara et al. highlighted Epstein-Barr virus (EBV)-specific polyfunctional CD8 T lymphocyte profiles as predictive biomarkers for infection control following pediatric liver transplantation. Similarly, Zavaglio et al. linked robust HCMV-specific T cell responses to spontaneous HCMV clearance in kidney transplant recipients.

Mele et al. described proof-of-concept for a novel whole-blood interferon-γ release assay for detecting and evaluating HCMV-specific CD4 T lymphocytes to improve HCMV infection management in immunocompromised patients—a major concern in transplantation medicine.

Additional work by Cuesta-Martín de la Cámara et al. aimed to identify immunological biomarkers as risk factors for opportunistic infection in pediatric liver transplantation. While significant predictive immunological markers could not be identified for early post-transplant infections, late-onset infections appeared connected with T-cell lymphopenia and hypogammaglobulinemia.

These studies collectively underscore the potential of threatening opportunistic infections (4, 5).

## Antibodies and cell-based advanced therapies

Anti-thymocyte globulin (ATG) is widely utilized in HCT for GVHD prophylaxis, available primarily as ATG-Fresenius (ATG-F) and ATG-Thymoglobulin (ATG-T). Determining optimal formulation and dosing remains challenging due to the need to balance effective GVHD prevention with associated infection risks. Falicovich et al. compared low-dose ATG-F to standard-dose ATG-T in unrelated HCT recipients, finding comparable GVHD incidence, overall survival, and non-relapse mortality. However, ATG-T recipients experienced significantly higher EBV reactivation rates, highlighting formulation-specific risks.

Mesenchymal stromal cells (MSCs) represent a promising therapeutic strategy due to their immunomodulatory capabilities (6). Liang et al. investigated human liver-derived MSCs (L-MSCs) in murine renal ischemia models, demonstrating their superiority in reducing inflammation and enhancing macrophage polarization toward anti-inflammatory and reparative phenotypes compared to bone marrow (BM-MSCs) and adipose MSC (A-MSCs). These findings highlight the importance of MSC source selection for maximizing therapeutic efficacy.

Clinical validation was provided by Hendriks et al., who conducted a phase Ib trial of allogeneic BM-MSC infusion in kidney transplant recipients. They reported MSC therapy safety and identified minor immune cell changes post-infusion. Notably, MSC infusions induced distinct, transient B and T cell phenotypes (CD11b^+^CD11c^+^Ki-67^+^), suggesting acute immune modulation. While the clinical relevance of these transient cells requires further investigation, their emergence underscores the rapid immunological influence of MSC treatments.

Wang et al. reviewed emerging biotechnological approaches, introducing the potential of allogeneic CAR virus-specific T cells (CAR-VST) designed to target both malignancy and infection without inducing GVHD.

Li et al. described a compelling strategy applied to a leukemia patient who relapsed after umbilical cord blood transplantation (UCBT) and was subsequently treated with CD19 CAR-T cells derived from the patient himself. The patient with refractory disease at CAR-T infusion is still in remission more than six years post-therapy.

Montagna et al. focused on establishing a Phase I/II clinical trial evaluating safety and preliminary efficacy of cell-based advanced therapy. Their proposed trial results from extensive preclinical studies investigating donor-derived cytotoxic T lymphocytes to prevent leukemia relapse in pediatric patients undergoing haploidentical HCT, a highly relevant and innovative concept addressing clear clinical needs.

## Conclusion

This Research Topic captures the dynamic state of alloimmunity research, from fundamental mechanistic studies to innovative clinical applications. The contributions highlight how technological advances, including single-cell analytics, spatial biology, and computational modeling, provide unprecedented insights into alloimmune complexity. These developments promise to advance our understanding and treatment of transplant-related complications while improving long-term patient outcomes.

## References

[1] Velardi A Ruggeri L Moretta A Moretta L. NK cells: a lesson from mismatched hematopoietic transplantation. Trends Immunol. (2002) 23:438–44. doi: 10.1016/s1471-4906(02)02284-6, PMID: 12200065

[2] Locatelli F Pende D Falco M Della Chiesa M Moretta A Moretta L. NK cells mediate a crucial graft-versus-leukemia effect in haploidentical-HSCT to cure high-risk acute leukemia. Trends Immunol. (2018) 39:577–90. doi: 10.1016/j.it.2018.04.009, PMID: 29793748

[3] Van Rood JJ Loberiza FR Jr Zhang MJ Oudshoorn M Claas F Cairo MS. Effect of tolerance to noninherited maternal antigens on the occurrence of graft-versus-host disease after bone marrow transplantation from a parent or an HLA-haploidentical sibling. Blood. (2002) 99:1572–7. doi: 10.1182/blood.v99.5.1572, PMID: 11861270

[4] Fishman JA. Infection in solid-organ transplant recipients. N Engl J Med. (2007) 357:2601–14. doi: 10.1056/NEJMra064928, PMID: 18094380

[5] Baldanti F Lilleri D Gerna G. Monitoring human cytomegalovirus infection in transplant recipients. J Clin Virol. (2008) 41:237–41. doi: 10.1016/j.jcv.2007.12.001, PMID: 18203657

[6] Le Blanc K Frassoni F Ball L Locatelli F Roelofs H Lewis I. Mesenchymal stem cells for treatment of steroid-resistant, severe, acute graft-versus-host disease: a phase II study. Lancet. (2008) 371:1579–86. doi: 10.1016/S0140-6736(08)60690-X, PMID: 18468541

